# Disseminated non-tuberculous mycobacterial infection caused by *Mycobacterium obuense* in an immunocompromised patient: a case report

**DOI:** 10.1186/s12879-023-08510-7

**Published:** 2023-08-08

**Authors:** Maiko Naito, Kentaro Fukushima, Shinsuke Kusakabe, Takaya Endo, Takayuki Shiroyama, Kika Ohira, Koji Azuma, Satoshi Tanizaki, Yumiko Yamamoto, Yuki Hosono, Yujiro Naito, Shinji Futami, Kotaro Miyake, Haruhiko Hirata, Yoshito Takeda, Atsushi Kumanogoh

**Affiliations:** 1 Department of Respiratory Medicine and Clinical Immunology, Graduate School of Medicine, Osaka University, 2-2 Yamadaoka, Suita, Osaka 565-0871 Japan; 2 Department of Hematology and Oncology, Graduate School of Medicine, Osaka University, 2-2 Yamadaoka, Suita, Osaka 565-0871 Japan

**Keywords:** Non-tuberculous mycobacteria, *Mycobacterium obuense*, Rapid growing mycobacteria, Disseminated infection, Miliary nodule

## Abstract

**Background:**

Mycobacterium *obuense* (M. *obuense*) is a rapidly growing mycobacterium (RGM) which has been considered nonpathogenic. Here, we report a case of disseminated non-tuberculous mycobacterial (NTM) infection caused by M. *obuense* in an immunocompromised patient.

**Case presentation:**

A 16-year-old boy was referred to our hospital due to acute myeloid leukemia. During the treatment of leukemia, the patient exhibited continuous fever, and diffuse miliary nodules with random distribution were found on chest computed tomography. Repeated examinations of bacterial culture tests revealed sputum and urine samples to be smear-positive for acid-fast bacillus, and blood culture from a peripherally inserted central catheter line showed the growth of NTM. The NTM species was identified as M. *obuense* by mass spectrometry and confirmed by genome sequencing. Combination therapy with amikacin, rifampicin, azithromycin, and moxifloxacin significantly improved the patient’s symptoms and radiological findings.

**Conclusion:**

We report a case of disseminated NTM infection caused by M. *obuense* for which combination anti-microbial therapy was effective. An immunocompromised host indwelling catheter is at risk of RGM bloodstream infections. Although relatively rare, M. *obuense* may be considered as a potential pathogen causing infectious diseases, especially in high-risk patients.

## Background

The prevalence of non-tuberculous mycobacterial (NTM) disease is increasing worldwide and is becoming a serious public health concern [1, 2]. Although more than 180 species have been discovered, only a few have been reported to be pathogenic [3]. The most common causative organisms of human infections are the slowly growing mycobacteria *Mycobacterium avium* complex (MAC), followed by *Mycobacterium kansasii* (M. *kansasii*) and the rapidly growing mycobacterium (RGM) [4]. RGM are characterized by visible growth on solid media within 7 days [5, 6]. M. *abscessus*, M. *chelonae*, and M. *fortuitum* are the most frequently encountered subspecies of RGM. They are associated with a wide range of diseases, such as skin and soft tissue infection, pulmonary infection, bloodstream infection, and disseminated infection [7]. M. *obusense*, a species of RGM, was considered nonpathogenic until recently [8–10].

Here, we report a case of disseminated NTM caused by M. *obuense* in an immunocompromised patient.

## Case presentation

A 16-year-old boy was referred to our department due to persistent high fever and an abnormal chest shadow.

Four months earlier, he had been diagnosed with RUNX1-RUNX1T1-positive acute myeloid leukemia (AML) (WHO classification 5th ed). RUNX1-RUNX1T1 is a fusion oncogene resulting from the chromosomal translocation t(8;21) and plays a crucial role in AML [11]. After one course of induction therapy (idarubicin 12 mg/m^2^ day1–3, cytarabine 100 mg/m^2^ day1–7), hematological complete remission (CR) was achieved, and the patient underwent consolidation therapy with a high-dose cytarabine (HDAC) regimen (cytarabine 2 g/m^2^ q12h day1–5). During the second course of HDAC therapy, the patient developed febrile neutropenia and was empirically treated with cefepime and teicoplanin. Later, blood culture showed *Corynebacterium striatum*. Despite the continuation of antibiotics and conversion to negative blood cultures, the patient consistently had high grade fever (temperature 40.1℃ axillary) and increased levels of inflammatory marker (C-reactive protein 12.85 mg/dL).

**Fig. 1:**
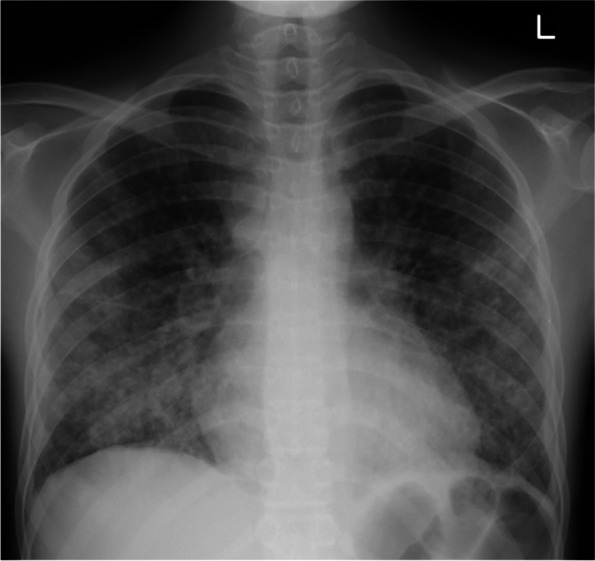
Chest radiograph showing lungs with bilateral miliary nodules

**Fig. 2:**
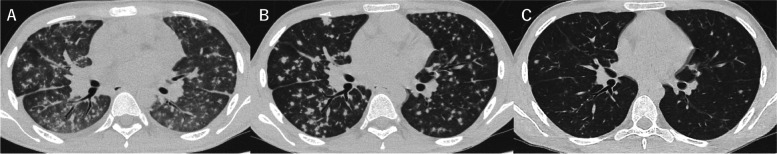
Chest CT at the time of consultation showing multiple small nodules with random distribution (**a**). After nadir period, pulmonary nodules enlarged (**b**). After treatment, lung nodules almost vanished (**c**)

**Fig. 3:**
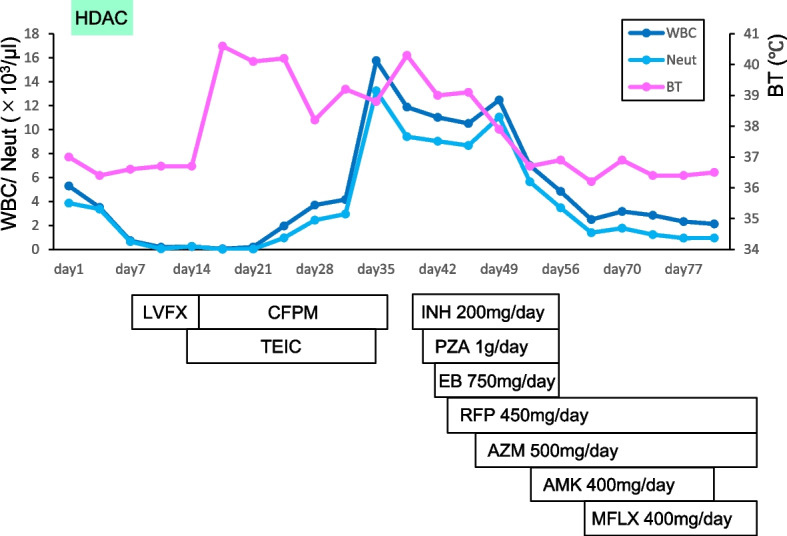
Clinical course of the patient. Fever and white blood cells decreased after the initiation of anti-tuberculosis treatment. HDAC: high-dose cytarabine, WBC: white blood cell, Neut: neutrophil, BT: body temperature, LVFX: levofloxacin, CFPM: cefepime, TEIC: teicoplanin, INH: isoniazid, PZA: pyrazinamide, EB: ethambutol, RFP: rifampicin, AZM: azithromycin, AMK: amikacin, MFLX: moxifloxacin

At the time of consultation, the patient showed leukopenia due to chemotherapy, with white blood cell count 200/μL, neutrophil count 20/μL, and lymphocyte count 30/μL. Chest radiography showed bilateral miliary nodules (Fig. 1) and chest computed tomography (CT) showed multiple diffuse small pulmonary nodules with random distribution (Fig. 2a). Miliary tuberculosis, disseminated infection, and malignancy (lung lesion of leukemia, lung cancer, and metastases of other cancers) was considered in the differential diagnosis from these radiological findings. Notably, the patient tested negative for human immunodeficiency virus (HIV). The cultures of three sputum specimens, lung biopsy specimens, blood, urine, and bone marrow fluid were all negative, although the acid-fast bacillus (AFB) were found in one of both the sputum and urine samples. Both Tb-PCR and 8 weeks culture was negative in these samples. Transbronchial lung biopsy showed neutrophil and lymphocyte infiltration in the alveolar space and thickened alveolar septums with no malignant findings. No causative mycobacteria, fungus, and bacteria were found in these lung biopsy specimens by staining and tissue culture. After recovery from the nadir period, as the neutrophils and lymphocytes increased, the small nodules found on chest CT enlarged and were accompanied by discrete hypoxemia (Fig. 2b). As we could not completely rule out the possibility of miliary tuberculosis, empiric anti-tuberculosis treatment (isoniazid 200 mg/day, pyrazinamide 1 g/day, ethambutol 750 mg/day, and rifampicin 450 mg/day) was initiated. After the initiation of the treatment, the patient’s clinical symptoms gradually improved. The fever and inflammatory markers decreased, and oxygen administration became unnecessary (Fig. 3). Subsequently, 7 weeks after chemotherapy initiation, a blood culture from a peripherally inserted central catheter (PICC) line showed the growth of RGM. The PICC was inserted 3 weeks before the consultation. Mass spectrometric analysis by MALDI TOF–MS (Bruker Corp., Massachusetts, USA) revealed that the cultured mycobacterium was M. *obuense*. Susceptibility to antimicrobial drugs was tested (Table 1), and the identification of M. *obuense* was confirmed by gene sequencing of 16 s rRNA. Therefore, the PICC was removed and intravenous amikacin 400 mg/day with a combination of oral medication (rifampicin 450 mg/day, azithromycin 500 mg/day, and moxifloxacin 400 mg/day) was initiated. After 4 weeks of combination therapy, the patient’s clinical symptoms and chest CT findings showed significant improvement (Fig. 2c), and the patient was discharged. 14 weeks have passed after discharge, and the patient is still continuing oral medication of rifampicin, azithromycin, and moxifloxacin at the outpatient department without relapse or any major adverse events.

**Table 1:** Antimicrobial drug susceptibility for *Mycobacterium obuense*

Drug	Susceptibility	MIC(μg/ml)
Clarithromycin	S	2
Amikacin	S	< 4
Imipenem	S	2
Faropenem	I	16
Levofloxacin	S	< 1
Moxifloxacin	S	< 0.25
Azithromycin	I	4
Sulfamethoxazole-Trimethoprim	S	< 0.25
Doxycycline	R	8
Meropenem	R	32
Linezolid	S	2
Clofazimine	NA	0.13
Sitafloxacin	S	<0.25

*S* Susceptible, *I* Intermediate, *R* Resistant, *NA* Not applicable

## Discussion and conclusion

Here, we present a case of disseminated M. *obuense* infection in an immunocompromised adolescent. In addition to an immunocompromised state, the prolonged used of vascular catheters increased the risk of RGM catheter-related bloodstream infections (CRBSI). To the best of our knowledge, this is the first report of a disseminated infection with miliary pulmonary nodule caused by M. *obuense*.

M. *obuense* was first described as a rapidly growing scotochromogenic mycobacterium isolated primarily from soil in 1971 in Obu, Japan [8]. M. *obuense* is a catalase-positive, peroxidase-negative bacillus that degrades salicylates and forms a black products [12]. Similar to other species of NTM, M. *obuense* exhibits two morphotypes in culture: smooth and rough variants. In smooth variants, the cell wall of M. *obuense* contains long-chain saturated fatty acids which enable to colonize the environment and are responsible for pleomorphisms [13]. Because it has been considered non-pathogenic, a heat-killed suspension of M. *obuense* has been investigated as a potential treatment for melanoma, pancreatic cancer, and colorectal cancer owing to its ability to stimulate anti-tumor T cell responses [14, 15]. However, this species was later isolated from sputum sample of a patient with a history of non–small cell lung cancer presenting with patchy lung infiltrates [16]. Additionally, a case of pneumonia with bacteremia caused by M. *obuense* in a patient with diabetes mellitus and chronic kidney disease [10], and a case of cutaneous infection after an insect bite in an immunocompetent patient has been reported [9], indicating its potential pathogenicity. To our knowledge, 3 cases presented above are the only available reports of M. *obuense* infection in the world, therefore, making our report rare and valuable.

As M. *obuense* is uncommon and very rare as an infection-causing bacterium, the identification of this species requires advanced examination. In our case, we performed mass spectrometry to identify M. *obuense* and confirmed the result by 16 s rRNA sequencing. Because it is difficult to identify M. *obuense* in a standard examination such as DNA-DNA hybridization, M. *obuense* infection might be overlooked in infectious diseases caused by unidentified RGM.

Disseminated disease due to NTM most commonly occurs in patients with advanced HIV infection with very low CD4^+^ T cell counts [17–19]. The average CD4^+^ T cell count at presentation has usually been less than 25 /μL in series of patients with confirmed disseminated NTM disease. All persons with CD4^+^ T cells lower than 50/μL are at risk of disseminated NTM, with the risk increasing with progressively fewer numbers of cells [18–21]. Although rare, dissemination of NTM in patients without HIV infection has been reported in immunosuppressed patients with organ transplantation, use of immunosuppressants, and cancer including leukemia [19, 22, 23]. Interestingly, while majority of disseminated NTM infections in patients with HIV are caused by MAC, RGM species are most commonly found in non-HIV patients [23, 24]. In the present case, an immunocompromised state due to leukemia and chemotherapy were likely risk factors for disseminated NTM infection. At the time of consultation, the patient’s total lymphocyte cell count was 30/μL, suggesting that CD4 + T cell was less than 30/μL, which is a high risk of disseminated infection.

Almost all RGM species can form biofilms and cause CRBSI [25, 26]. Because RGM embedded in dense biofilms become resistant to antimicrobial treatment, catheter removal and treatment with at least two active antimicrobial agents are generally recommended [27, 28]. The duration of treatment for CRBSI caused by RGM differs between studies, nonetheless, at least 4 weeks of a combination antimicrobial regimen was associated with favorable outcomes in immunocompetent patients [27, 29]. However, it is reported that non-HIV infected immunocompromised disseminated NTM patients with blood culture isolates should be treated with antimycobacterial drugs for at least 6 to 12 months after immune restoration [19]. In the present case, we intend to continue oral combination antimicrobial treatment with rifampicin, azithromycin, and moxifloxacin for at least 6 months. RGM identified in routine blood cultures are usually regarded as true pathogens. However, because these organisms are ubiquitous in the environment, we should be aware of contamination. In our case, although blood culture was positive in only one sample, the AFB was positive in multiple organ derived samples. Moreover, the fact that anti-microbial drugs improved the clinical symptoms and miliary nodules seen on chest CT indicated that the disease was caused by disseminated mycobacterium infection. In addition, the worsening of chest CT findings occurring at the recovery of the nadir indicates a paradoxical reaction of disseminated NTM infection caused by an augmented immune system.

In conclusion, we experienced a case of disseminated NTM infection caused by M. *obuense*. An immunocompromised state and the use of catheters, including PICC, are risk factors for disseminated NTM infections. Although M. *obusene* infection remains rare, it should be considered a potential pathogen when detected in culture.

## Data Availability

The datasets supporting the conclusions of this article are included within the article.

## Abbreviations

AFB: Acid-fast bacillus
CT: Computed tomography
CR: Complete remission
CRBSI: Catheter-related bloodstream infection
HDAC: High-dose cytarabine
HIV: Human immunodeficiency virus
MAC: *Mycobacterium avium* complex
M. *obuense*: *Mycobacterium obuense*
NTM: Non-tuberculous mycobacterium
PICC: Peripherally inserted central catheter
RGM: Rapidly growing mycobacterium

## References

[1] Dahl VN, Mølhave M, Fløe A, van Ingen J, Schön T, Lillebaek T, Andersen AB, Wejse C (2022). Global trends of pulmonary infections with nontuberculous mycobacteria: a systematic review. Int J Infect Dis.

[2] Brode SK, Daley CL, Marras TK (2014). The epidemiologic relationship between tuberculosis and non-tuberculous mycobacterial disease: a systematic review. Int J Tuberc Lung Dis.

[3] Cowman S, van Ingen J, Griffith DE, Loebinger MR (2019). Non-tuberculous mycobacterial pulmonary disease. Eur Respir J.

[4] Koh WJ. Nontuberculous mycobacteria—overview. Microbiol Spectrum. 2017;5(1):TNMI7–0024–2016. 10.1128/microbiolspec.tnmi7-0024-2016 PMC11687458 28128073

[5] Runyon EH (1959). Anonymous mycobacteria in pulmonary disease. Med Clin North Am.

[6] Runyon EH (1970). Identification of mycobacterial pathogens utilizing colony characteristics. Am J Clin Pathol.

[7] De Groote MA, Huitt G (2006). Infections due to rapidly growing mycobacteria. Clin Infect Dis.

[8] Tsukamura M, Mizuno S (1971). Mycobacterium obuense, a rapidly growing scotochromogenic mycobacterium capable of forming a black product from p-aminosalicylate and salicylate. J Gen Microbiol.

[9] Boyd AS (2018). Cutaneous infection with Mycobacterium obuense. Int J Mycobacteriol.

[10] Luis BAL, Díaz-Lomelí P, Gómez-Albarrán LP, Martínez-Gamboa A, Ponce-de-León A (2019). Mycobacterium obuense Bacteremia in a Patient with Pneumonia. Emerg Infect Dis.

[11] Gilliland DG, Jordan CT, Felix CA. The molecular basis of leukemia. Hematol Am Soc Hematol Educ Program. 2004(1):80–97. 10.1182/asheducation-2004.1.80 15561678

[12] Tsukamura M, Mizuno S, Tsukamura S (1981). Numerical Analysis of Rapidly Growing, Scotochromogenic Mycobacteria, Including Mycobacterium obuense sp. nov., nom. rev., Mycobacterium rhodesiae sp. nov., nom. rev., Mycobacterium aichiense sp. nov., nom. rev., Mycobacterium chubuense sp. nov., nom. rev., and Mycobacterium tokaiense sp. nov., nom. rev. Int J Syst Bacteriol.

[13] Agustí G, Astola O, Rodríguez-Güell E, Julián E, Luquin M (2008). Surface spreading motility shown by a group of phylogenetically related, rapidly growing pigmented mycobacteria suggests that motility is a common property of mycobacterial species but is restricted to smooth colonies. J Bacteriol.

[14] Stebbing J, Dalgleish A, Gifford-Moore A, Martin A, Gleeson C, Wilson G, Brunet LR, Grange J, Mudan S (2012). An intra-patient placebo-controlled phase I trial to evaluate the safety and tolerability of intradermal IMM-101 in melanoma. Ann Oncol.

[15] Dalgleish AG, Stebbing J, Adamson DJ, Arif SS, Bidoli P, Chang D, Cheeseman S, Diaz-Beveridge R, Fernandez-Martos C, Glynne-Jones R (2016). Randomised, open-label, phase II study of gemcitabine with and without IMM-101 for advanced pancreatic cancer. Br J Cancer.

[16] Greninger AL, Cunningham G, Hsu ED, Yu JM, Chiu CY, Miller S (2015). Draft genome sequence of mycobacterium obuense Strain UC1 isolated from patient sputum. Genome Announc.

[17] Horsburgh CR (1996). Epidemiology of disease caused by nontuberculous mycobacteria. Semin Respir Infect.

[18] Horsburgh CR, Gettings J, Alexander LN, Lennox JL (2001). Disseminated Mycobacterium avium complex disease among patients infected with human immunodeficiency virus, 1985–2000. Clin Infect Dis.

[19] Griffith DE, Aksamit T, Brown-Elliott BA, Catanzaro A, Daley C, Gordin F, Holland SM, Horsburgh R, Huitt G, Iademarco MF (2007). An official ATS/IDSA statement: diagnosis, treatment, and prevention of nontuberculous mycobacterial diseases. Am J Respir Crit Care Med.

[20] Horsburgh CR, Selik RM (1989). The epidemiology of disseminated nontuberculous mycobacterial infection in the acquired immunodeficiency syndrome (AIDS). Am Rev Respir Dis.

[21] Nightingale SD, Byrd LT, Southern PM, Jockusch JD, Cal SX, Wynne BA (1992). Incidence of Mycobacterium avium-intracellulare complex bacteremia in human immunodeficiency virus-positive patients. J Infect Dis.

[22] Kiehn TE, White M (1994). Mycobacterium haemophilum: an emerging pathogen. Eur J Clin Microbiol Infect Dis.

[23] Chetchotisakd P, Mootsikapun P, Anunnatsiri S, Jirarattanapochai K, Choonhakarn C, Chaiprasert A, Ubol PN, Wheat LJ, Davis TE (2000). Disseminated infection due to rapidly growing mycobacteria in immunocompetent hosts presenting with chronic lymphadenopathy: a previously unrecognized clinical entity. Clin Infect Dis.

[24] Ingram CW, Tanner DC, Durack DT, Kernodle GW, Corey GR (1993). Disseminated infection with rapidly growing mycobacteria. Clin Infect Dis.

[25] Esteban J, Martín-de-Hijas NZ, Kinnari TJ, Ayala G, Fernández-Roblas R, Gadea I (2008). Biofilm development by potentially pathogenic non-pigmented rapidly growing mycobacteria. BMC Microbiol.

[26] El Helou G, Viola GM, Hachem R, Han XY, Raad II (2013). Rapidly growing mycobacterial bloodstream infections. Lancet Infect Dis.

[27] El Helou G, Hachem R, Viola GM, El Zakhem A, Chaftari AM, Jiang Y, Tarrand J, Raad II (2013). Management of rapidly growing mycobacterial bacteremia in cancer patients. Clin Infect Dis.

[28] Redelman-Sidi G, Sepkowitz KA (2010). Rapidly growing mycobacteria infection in patients with cancer. Clin Infect Dis.

[29] Mizusawa M, Vindenes T, Buckley S, Armstrong C (2020). A case series of rapidly growing mycobacterial catheter-related bloodstream infections among immunocompetent patients. J Clin Tuberc Other Mycobact Dis.

